# Sedentary Behaviour and Physical Activity in South Asian Women: Time to Review Current Recommendations?

**DOI:** 10.1371/journal.pone.0058328

**Published:** 2013-03-05

**Authors:** Indu Waidyatilaka, Pulani Lanerolle, Rajitha Wickremasinghe, Sunethra Atukorala, Noel Somasundaram, Angela de Silva

**Affiliations:** 1 Department of Biochemistry and Molecular Biology, Faculty of Medicine, University of Colombo, Sri Lanka; 2 Department of Public Health, Faculty of Medicine, University of Kelaniya, Ragama, Sri Lanka; 3 Endocrine Unit, National Hospital of Colombo, University of Colombo, Sri Lanka; 4 Department of Physiology, Faculty of Medicine, University of Colombo, Sri Lanka; The University of Hong Kong, Hong Kong

## Abstract

**Objective:**

Our aims were to describe activity and sedentary behaviours in urban Asian women, with dysglycaemia (diagnosed at recruitment), and without dysglycaemia and examine the relative contribution of these parameters to their glycaemic status.

**Methods:**

2800 urban women (30–45 years) were selected by random cluster sampling and screened for dysglycaemia for a final sample of 272 newly diagnosed, drug naive dysglycaemic and 345 normoglycaemic women. Physical activity and sedentary behaviours were assessed by the International Physical Activity Questionnaire (IPAQ). Demographic data, diet and anthropometry were recorded. Logistic regression analysis assessed contribution of all parameters to dysglycaemia and exposure attributable fractions were calculated.

**Results:**

The mean energy expenditure on walking (2648.5±1023.7 MET-min/week) and on moderate and vigorous physical activity (4342.3±1768.1 MET-min/week) for normoglycemic women and dysglycaemic women (walking;1046.4±728.4 MET-min/week, moderate and vigorous physical activity; 1086.7±1184.4 MET-min/week) was above the recommended amount of physical activity per week. 94.3% of women spent >1000 MET-minutes/week on activity. Mean sitting and TV time for normoglycaemic and dysglycaemic women were 154.3±62.8, 38.4±31.9, 312.6±116.7 and 140.2±56.5 minutes per day respectively. Physical activity and sedentary behaviour contributed to dysglycaemia after adjustment for family history, diet, systolic blood pressure and Body Mass Index. Exposure attributable fractions for dysglycaemia were; lower physical activity: 78%, higher waist circumference: 94%, and TV viewing time: 85%.

**Conclusions:**

Urban South Asian women are at risk of dysglycaemia at lower levels of sedentary behaviour and greater physical activity than western populations, indicating the need for re-visiting current physical activity guidelines for South Asians.

## Introduction

Type 2 diabetes mellitus has reached epidemic proportions in Asian countries, leading to significant increases in morbidity and mortality [1]. Preventing further escalation of this problem requires culturally appropriate interventions, taking into account physical activity patterns, dietary habits and other behaviours. The Diabetes Prevention Program, though not specifically aimed at Asians, had a small Asian sub population who were assessed; Asians had a greater risk reduction of diabetes mellitus with lifestyle intervention (71% vs. 51%), inclusive of physical activity, as compared to the white population [2]. Other studies focusing on Asians [3], [4], [5], have firmly established the role of physical activity in reducing risk of non communicable diseases [6], [7]. The current global guidelines on physical activity recommend 150 minutes of moderate intensity physical activity or 75 minutes of vigorous physical activity per week to achieve substantial health benefits [8]. In terms of energy expenditure this amounts to 500 to 1000 MET-minutes of physical activity per week [9], [10]. Recent data also suggests that in addition to reducing physical inactivity, sedentary behaviours maybe important in the aetiology of dysglycaemia and type 2 diabetes mellitus. Sedentary behaviour, a separate entity defined as engaging in activities at the resting level of energy expenditure which includes sleeping, sitting, lying down, computer time, and viewing television [11], its associations and role in diabetes mellitus are less well studied.

Two key studies, the U.S. National Health and Nutrition Examination Survey (NHANES) and the Australian Diabetes, Obesity and Lifestyle (AusDiab) study, demonstrated that total sedentary time was associated with increased cardiometabolic risk, independent of leisure time physical activity and adiposity [12], [13]. In another study, adverse effects of prolonged sitting have been shown even in those who met exercise guidelines [14]. Individual components of sedentary behaviour such as sitting time [15] and television (TV) viewing time have also been evaluated, with TV viewing time receiving the most attention since it is one of several common behaviours that involve prolonged sitting and has been linked to overweight and obesity [16], [17]. Average TV viewing time, the most common leisure time sedentary behaviour, is approximately between three hours in the UK and eight hours in the USA [18].

While it is known that for a given BMI, Asians have higher body fat [19], the relative effects of physical activity, sedentary behaviours, anthropometry and poor dietary practices on glycaemic status in this population are yet unclear. Some data indicates that urbanization in parts of Asia has lead to greater physical inactivity [20]. However, in Asian communities, time available for leisure time activity, both sedentary and physical, is often limited due to socioeconomic circumstances and Western models may not reflect current practices in many parts of Asia. Data on physical activity, walking time and sedentary behaviours such as sitting, TV viewing time, and their relative contribution to glycaemia in Asian populations living in Asia are scarce. Our aims were to describe activity and sedentary behaviours in urban Asian women, with dysglycaemia (diagnosed at recruitment), and without dysglycaemia and examine the relative contribution of these parameters to their glycaemic status.

## Materials and Methods

### Study Population

Participants in the study were women aged 30 to 45 years living in the Colombo Municipal Council area. A cluster was defined as the smallest administrative unit (Grama Niladhari division) within the Colombo Municipal Council area, which consists of 55 such units. From all the selected Grama Niladhari divisions, 51 women in the 30–45 year age range were randomly selected for screening in order to obtain the estimated 196 women with dysglycaemia required for the larger study with an allowance for drop outs. Glycaemic status was confirmed by HbA1c in all those who were recruited and women were grouped according to the American Diabetic Association 2012 classification [21]. All 272 dysglycaemics detected in the screening procedure were enrolled in the study. A further 345 normoglycaemic women were randomly selected from the entire screened sample with representation from all Grama Niladhari areas. The sample numbers in both groups were sufficient to detect differences reported in all variables in this study with over 90% power and an alpha error of 5%.Women who were pregnant or breast feeding, having an acute infection, on long term steroids or reporting significant weight loss were excluded from screening.

### Methodology

Demographic information of the study population, including family, past medical and drug histories and selected food related practices were obtained using an interviewer administered questionnaire. The International Physical Activity Questionnaire (IPAQ) validated for use in Sri Lanka was used to assess time (minutes) spent on moderate and vigorous physical activity, walking and time spent sitting on weekdays and weekends [22], [23]. Weekly time spent on each activity was calculated by multiplying frequency (number of days) and duration (minutes per day) of each activity on a typical day. All questions referred to the week immediately preceding the interview. In addition to the IPAQ, data on sedentary behaviours; time spent sitting at work, having meals, viewing TV, with family, during leisure time and any other periods of sitting were also collected. Time when the TV was switched on but other activities were carried out were excluded from the calculation of TV time.

Waist circumference was measured in standing position at the end of normal expiration, in the horizontal plane at the level of the narrowest point between the lower costal border and the iliac crest, to the nearest 0.1 cm using a non-stretchable measuring tape (Seca 200). All measurements were taken in duplicate and the mean was calculated. Height and weight were recorded while the women were standing barefoot. Height was measured to the nearest 0.1 cm using a Stadiometer (Seca 225, telescopic height measurement). Weight was measured to the nearest 0.1 kg using a calibrated electronic scale (Seca 813) and Body Mass Index (BMI) was calculated as weight/height ^2^. All measurements were taken by the same trained observer according to the International Society for Advancement of Kineanthropometry protocol [24]. Sitting blood pressure was recorded in duplicate (using a mercury sphygmomanometer) after at least 15 minutes rest. All measurements were taken on the left arm by the same trained observer. Venous blood samples were collected under aseptic conditions following an overnight fast (10–12 hours). HbA1C was assayed by High Performance Liquid Chromatography (NGSP certified and standardized to the Diabetes Control and Complications Trial assay) by Bio-Rad D-10 analyzer (France) and fasting blood glucose by the glucose oxidase method on a Hitachi 911 analyzer (Hitachi instruments, inc., USA) using reagents from Roche diagnostics. A food index was computed using three questions that represent unhealthy eating behaviours; eating while watching television, consumption of high fat food, and eating sweet/sugary foods. There were two response categories for the question on eating while viewing TV; two points were assigned to response “Yes” (snacking while watching TV), and 0 for response “No”. The two questions on fat and sugary foods was based on a Likert scale with four response categories ranging from “Always” to “Never”, with 2 and 1 points assigned for “Always” and “Almost always”, respectively, and 0 for the other responses.

### Statistical Analysis

All analyses were done using SPSS version 18.0. Metabolic Equivalents were calculated using IPAQ guidelines and expressed as MET-minutes/week. Means ± SDs were calculated for all continuous variables and the percentage of participants in each category was determined for all categorical variables. Proportion of women expending more than 1000 MET-minutes/week on physical activity was calculated. Analyses of variance for continuous variables and Chi-square test for trend for categorical variables were used to compare differences in groups. Spearman correlation coefficient was used to determine the correlation of HbA1c with all risk variables (sitting time, TV viewing time, walking MET-minutes, moderate and vigorous physical activity MET-minutes, systolic and diastolic blood pressure, waist circumference and BMI).

In order to dichotomize sitting and TV viewing time, walking MET-minutes and moderate and vigorous physical activity MET-minutes for logistic regression, cut off values were derived for identification of women with dysglycaemia using Receiver Operating Characteristics (ROC) curve analysis based on maximum sensitivities and specificities. Logistic regression analyses were carried out to assess risk. Odds ratios (OR) and 95% confidence intervals of OR and attributable fractions among exposed, calculated as ((OR-1)/OR), to estimate the risk of dysglycaemia using family history, food index, anthropometric indices, sedentary behaviours and moderate and vigorous physical activity and walking as independent variables assuming that controls are representative of the general population and that the prevalence of exposure is low.

### Ethics

The study protocol was approved by the Ethics Review Committee of the Faculty of Medicine of the University of Colombo, Sri Lanka. Procedures followed were in accordance with the Ethics standards of the responsible committee on human experimentation. All participants were informed about the study both verbally and in writing and written consent obtained. Women who were found to have diabetes were referred to the diabetic clinic of the tertiary hospital in Colombo which is managed by an endocrinologist. All women were given (and explained) the biochemical test results relating to their glycaemic and risk status. All newly diagnosed dysglycaemics were individually counselled and offered advice on diet and exercise.

## Results

Baseline characteristics of normoglycaemic women and newly diagnosed dysglycaemic women are given by glycaemic status in Table 1. Age, family income, employment status and educational level were similar in both groups. Newly diagnosed dysglycaemic women were more likely to have a family history of type 2 diabetes mellitus, a higher BMI and waist circumference, elevated blood pressures, spend significantly less time on moderate and vigorous physical activity and walking and more time on sedentary behaviour (Table 1).

**Table 1:** Selected characteristics of the study population.

Characteristic	Normoglycaemics (n = 345)	Newly diagnosed dysglycaemics (n = 272)
**Socio-demographic characteristics**		
Age (years)	37.30 (3.6)	37.89 (3.8)
Employed (%)	81.2%	75.7%
Family income per month (LKR^b^)	37,121(22,388)	35,321 (26,529)
Completed secondary education (%)	87.2%	82.7%
Family history of diabetes (%)	37.4%	57.4%^a^
**Physical activity**		
Moderate and vigorous physical Activity (MET-min/week)	4342.3 (1768.1)	1806.7 (1184.4)^a^
Walking (MET-min/week)	2648.5 (1023.7)	1046.4 (728.4)^a^
Proportion of women expending >1000 MET-min/week of physical activity (%)	100%	87.1%
**Sedentary behaviour**		
Sitting time (min/day)	154.3 (62.8)	312.6 (116.7)^a^
TV time (min/day)	38.4 (31.9)	140.2 (56.5)^a^
**Obesity indices**		
Waist circumference (cm)	70.3 (6.29)	81.1 (7.43)^a^
BMI (kg/m^2^)	22.09(3.51)	27.02 (4.51)^a^
**Blood pressure**		
Systolic blood pressure (mmHg)	117.6 (13.7)	135.7 (12.8)^a^
Diastolic blood pressure (mmHg)	72.6 (7.83)	81.0 (9.06)^a^
**Biochemistry**		
HbA1c (%)	5.3 (0.24)	7.9 (2.32)^a^
Fasting blood sugar (mg/dl)	85.0 (10.0)	149.6 (52.7)^a^

Continuous data are presented as mean (SD).

a Significantly different (p<0.001) from normoglycaemics.

b Conversion Rate 1 LKR = 0.0075 U.S Dollars.

Normoglycaemic women spent a mean of 2648.5±1023.7 MET-min/week on walking and a mean of 4342.3±1768.1 MET-min/week on moderate and vigorous physical activity. Women with dysglycaemia spent a mean of 1046.4±728.4 MET-min/week on walking and a mean of 1086.7±1184.4 MET-min/week on moderate and vigorous physical activity. 94.3% of women spent more than 1000 MET-minutes/week on walking and moderate and vigorous activity (global recommendation). Normoglycaemic women spent a mean of 154.3±62.8 minutes on sitting and 38.4±31.9 minutes on TV viewing per day while women with dysglycaemia spent a mean of 312.6±116.7 minutes on sitting and 140.2±56.5 minutes on viewing TV per day. Table 2 indicates correlation of dysglycaemia with moderate and vigorous physical activity, walking, sedentary behaviour, systolic blood pressure, waist circumference and BMI. Moderate and vigorous physical activity MET-minutes and walking were negatively correlated with HbA1c; sedentary behaviour, blood pressures and anthropometric indicators were positively correlated. In order to dichotomize variables for logistic regression, ROC curve analysis was used for moderate and vigorous physical activity MET-minutes, walking MET-minutes, sitting time and TV viewing time. ROC curve cut off values for dysglycaemia with optimal sensitivity and specificity are given in Table 3. ROC derived cut offs for walking and for moderate and vigorous intensity physical activity to identify women with dysglycaemia were greater than the recommended weekly energy expenditure on physical activity of 1000 MET min/week.

**Table 2:** Correlation of HbA1c with risk factors of dysglycaemia and physical activity.

Variable	Spearman correlation coefficient	*p* value
**Sedentary behaviour**		
Sitting time (minutes/day)	0.659	<0.001
TV time (minutes/day)	0.723	<0.001
**Physical activity**		
Walking (MET-min/week)	−0.684	<0.001
Moderate and vigorous physical activity (MET-min/week)	−0.650	<0.001
Walking time (minutes/day)	−0.688	<0.001
**Blood pressure**		
Systolic Blood Pressure (mmHg)	0.582	<0.001
Diastolic Blood Pressure (mmHg)	0.438	<0.001
**Obesity indices**		
Waist circumference (cm)	0.665	<0.001
BMI (kg/m^2^)	0.514	<0.001

**Table 3:** Receiver Operating Characteristics curve cut off values for dysglycaemia with sensitivity and specificity.

Variable	Cut-off	AUC	95% CI	Sensitivity	Specificity
Sitting time	185 min/day	0.91	0.88–0.93	85%	77%
TV viewing time	85 min/day	0.94	0.92–0.96	85%	87%
Walking	1435.5 MET-min/week	0.92	0.90–0.94	90%	81%
Moderate and vigorous physical activity	2640.0 MET-min/week	0.91	0.88–0.93	84%	85%

AUC – area under the curve.

Correlates of dysglycaemia in logistic regression analysis are shown in Table 4. Two models were derived. In model A, family history, food index, systolic blood pressure, BMI, waist circumference and the dichotomized variables (walking MET-minutes, moderate and vigorous physical activity MET-minutes, sitting time and TV viewing time), were used as independent variables (Table 4). In this preliminary model, moderate and vigorous physical activity MET-minutes, walking MET-minutes, TV viewing time, food index, and waist circumference were significantly associated with dysglycaemia after controlling for each other. In model B, systolic blood pressure, BMI and sitting time were excluded and an interaction term between waist circumference and TV viewing time was included (Table 4). Excepting family history of diabetes mellitus, all other variables including the interaction term were significantly associated with dysglycaemia. Females expending <1435.3 MET-minutes/week on walking and <2640.0 MET-minutes/week in moderate and vigorous physical activity were four times as likely to be dysglycaemic when compared to females with higher physical activity, giving an attributable fraction of 78% after adjusting for other variables. Women viewing TV for >85 minutes were six times as likely to be dysglycaemic when compared to those spending less time viewing TV; 85% of dysglycaemics could attribute their condition to viewing TV >85 minutes a day after adjusting for other variables. Women with a waist circumference >80 cm were 16 times as likely to be dysglycaemic when compared to females having waist circumference <80 cm; 94% of dysglycaemics could attribute their condition to having a waist circumference of over 80 cm after adjusting for other variables. The interaction between TV viewing time and waist circumference indicated that risk of dysglycaemia in women who watch TV >85 minutes and those having a waist circumference >80 cm was not a synergistic function of the individual risks of waist circumference and TV viewing. No significant interactions were found between moderate and vigorous physical activity, TV viewing time and food index.

**Table 4:** Models of regression analysis using dysglycaemia as the dependent variable.

	Regression coefficient	P value	OR	Attributable Fraction among exposed	95% CI
**Model A** a					
Family history^c^	0.32	0.282	1.38	0.28	0.77–2.49
Food index^d^	0.21	0.019	1.23	0.19	1.03–1.47
Systolic blood pressure^e^	0.05	0.897	0.95	−0.05	0.47–1.95
Walking MET-minutes/week^f^	1.51	<0.001	4.56	0.78	2.37–8.77
Moderate and vigorous physical activity MET-minutes/week^g^	1.41	<0.001	4.10	0.76	2.08–8.05
Sitting time^k^	0.36	0.306	1.43	0.29	0.72–2.82
TV viewing time^m^	1.44	<0.001	4.23	0.76	2.13–8.41
Waist circumference^n^	1.99	<0.001	7.34	0.86	3.06–17.62
BMI	0.04	0.904	1.05	0.04	0.50–2.17
**Model B** b					
Family history^c^	0.27	>0.05	1.31	0.23	0.73–2.34
Food index^d^	0.18	<0.05	1.20	0.16	1.01–1.42
Walking MET-minutes/week^f^	1.52	<0.001	4.58	0.78	2.40–8.76
Moderate and vigorous physical activity MET-minutes/week^g^	1.56	<0.001	4.78	0.79	2.45–9.28
TV viewing time^m^	1.86	<0.001	6.44	0.84	3.08–13.48
Waist circumference^n^	2.77	<0.001	15.99	0.94	5.60–45.65
Waist*TV viewing time	−1.37	0.05	0.26	−2.92	0.06–1.03

a Goodness of fit χ ^2^ = 0.044 (Hosmer and Lemeshow Test).

b Goodness of fit χ ^2^ = 0.154 (Hosmer and Lemeshow Test).

c Reference group is persons without a family history of diabetes.

d Reference group is persons with food index = 0.

e Reference group is persons with systolic blood pressure less than 140 mmHg.

f Reference group is persons exerting more than 1435.5 MET-minutes/week on walking.

g Reference group is persons exerting more than 2640.0 MET-minutes/week on moderate and vigorous physical activity.

k Reference group is persons whose sitting time was less than 185 min/day.

m Reference group is persons whose TV viewing time was less than 85 min/day.

n Reference group is persons whose waist circumference was less than 80 cm.

## Discussion

Our results indicate that most women spent more than 1000 MET-minutes/week on walking, moderate and vigorous physical activities, with 500–1000 MET minutes/week being the recommended energy expenditure on physical activity for health benefit, based on the global guidelines [9], [10]. Physical activity levels for normoglycaemic and dysglycaemic groups when taken separately also indicated higher mean levels of energy expenditure on physical activity than the recommendations, while both normoglcaemic and dysglycaemic groups showed low means for sedentary behaviour. These urban South Asian women appear to be at risk of dysglycaemia at a higher level of physical activity and lower levels of sedentary behaviour than western populations as indicated by the ROC derived cut offs to identify women with dysglycaemia for walking, and moderate and vigorous physical activity and TV viewing time. Majority of both dysglycaemic and normoglycaemic women led an active lifestyle through mostly non leisure time activities and low levels of sedentary behaviours. The high attributable fraction among exposed for high waist circumference indicates the role of overweight in a population that is physically active and the contribution of diet to dysglycaemia was seen through undesirable food practices.

Objective data on glycaemic status and activity patterns in an urban community of women was possible through our study design, where women with prediabetes and diabetes were newly diagnosed at recruitment, with women being unaware of their condition, through a community based random screening method. Since the women with dysglycaemia in our study population were only diagnosed at recruitment, and drug naïve, their lifestyles were unlikely to be influenced by their glycaemic status, and were reflective of a healthy population.

The ROC derived cut off for walking (1435.5 MET-minutes/week) and for moderate and vigorous physical activity (2640 MET-minutes/week) indicates that these women are dysglycaemic at a high level of physical activity. On average, time spent on walking and moderate and vigorous activity by women is much higher than reported elsewhere [25] and is not predominantly as a mode of exercise but as part of their daily routine chores. Women also spent less time on sedentary behaviours than commonly reported from Western populations [13]. Data from our study population suggest an active urban lifestyle, even when compared to data from other developing countries which report higher levels of physical inactivity linked to urbanization [26], [27], [28]. In contrast to Western data, where average TV viewing time is approximately 3–8 hours, [18], most women in our study spent just over an hour each day on TV viewing. The ROC derived cut off of 85 minutes TV watching time, with 85% sensitivity and 87% specificity, is more a reflection of how even low rates of sedentary behaviours affect dysglycaemia in this study population, rather than a cut off to be used in public health interventions.

Our data, points to the possibility, that despite achieving adequate physical activity in terms of global recommendations, even such levels maybe inadequate for urban South Asian women for health benefit. While the current cut offs may be questioned, adequacy of the classifications used both in the current methods of assessment such as IPAQ and also in the guidelines themselves is another area that needs further thought. There may be a need to revisit the classifications which are currently limited and do not allow non leisure activities to be studied in adequate detail in order to assess which types of non leisure activities may or may not confer protection against chronic disease. It is possible that within these physical activity and sedentary behaviour patterns, obesity may contribute to dysglycaemia, as shown by the high attributable risk of waist circumference in our study. Undesirable food behaviours as indicated by a food index also contributed to dysglycaemia in this study population. Though snacking behaviour as a reflection of dietary practice has its limitations, it has been shown to be linked to obesity [29]. However, data on total energy intake may have been better able to identify the role of diet in this sample of women. Our findings highlight the need to look beyond overall activity and sedentary behaviours in prevention of both prediabetes and diabetes. While diet and genetics may play a role, the issue of how much physical activity is preventive and how low sedentary behaviours must be to prevent dysglycaemia in South Asians needs attention. In this context, our findings support the concern that although research has shown benefits of the global guideline of 150 minutes/week of exercise, questions need to be raised regarding the adequacy of the current cut off, its dose intensity and duration in the presence of a possible diversity in response [26].

In summary, this data adds evidence to the limited data pool on contributory factors to dysglycaemia among South Asian women. Given that prevalence of dysglycaemia is rising in South Asian countries [30], several questions are raised by these findings; are the current guidelines on physical activity adequate for South Asian populations? Can we quantify total physical activity and sedentary behaviour in terms that can be translated into recommendations? How much of a relative role does diet and genetics play in South Asians? Given the limitations of cross sectional design we show that despite active lifestyles when compared to the West, South Asian women are at risk of dysglycaemia at lower levels of sedentary behaviour and more physical activity, to which overweight and undesirable food habits contribute, these effects being independent of the risk associated with having a family history of dysglycaemia.
